# MIG and TIG Joining of AA1070 Aluminium Sheets with Different Surface Preparations

**DOI:** 10.3390/ma15020412

**Published:** 2022-01-06

**Authors:** Elisa Fracchia, Jana Bidulská, Róbert Bidulský, Marco Actis Grande

**Affiliations:** 1 Department of Management and Production Engineering (DIGEP), Polytechnic of Turin, Corso Duca degli Abruzzi 24, 10129 Torino, Italy; elisa.fracchia@polito.it; 2 EPMA PM R&D Centre, Faculty of Materials, Metallurgy and Recycling, Technical University of Kosice, Park Komenskeho 10, 040 01 Kosice, Slovakia; 3 Asian Innovation Hub, Budulov 174, 045 01 Moldava nad Bodvou, Slovakia; robert.bidulsky@asihub.org; 4 Department of Applied Science and Technology (DISAT), Polytechnic of Turin, Viale T. Michel 5, 15121 Alessandria, Italy; marco.actis@polito.it

**Keywords:** AA1070, TIG welding, MIG welding, micro-hardness, tensile stress, microstructures

## Abstract

In this work, AA1070 aluminium alloy sheets are joined using TIG and MIG welding after three different edge preparations. Shearing, water jet and plasma-cut processes were used to cut sheets, subsequently welded using ER5356 and ER4043 filler metals for TIG and MIG, respectively. Mechanical properties of the obtained sheets were assessed through tensile tests obtaining a relation between sheet preparation and welding tightness. Micro-hardness measures were performed to evaluate the effects of both welding and cutting processes on the micro-hardness of the alloy, highlighting that TIG welding gives rise to inhomogeneous micro-hardness behaviour. After tensile tests, surface fractures were observed employing scanning electron microscopy to highlight the relation between tensile properties and edge preparations. Fractures show severe oxidation in the water jet cut specimens, ductile fractures and gas porosities.

## 1. Introduction

The applications of aluminium alloys are mainly related to their excellent mechanical properties, corrosion resistance and lightweight [1]. Furthermore, Al alloys have a low melting point and high thermal conductivity [2]. These properties induce manufacturers to use aluminium alloys for many different purposes.

Furthermore, aluminium alloys are weldable by arc welding, resistance welding and friction stir welding processes [3]. In particular, tungsten inert gas (TIG) or metal inert gas (MIG) are commonly used. A welded joint, in general, consists of three regions: the base metal (BM), the heat-affected zone (HAZ) and the welded metal (WM). Various parameters affect the welding of aluminium alloys, such as the thermal cycle, the specific welding process, the metal’s thickness, the metal’s thermal conductivity and the possible preheating before starting welding. In particular, various precautions, such as proper edge preparation and careful choice of filler metal, are crucial to realize a metallurgical joint free of defects.

As reported in the ASM handbook [4], aluminium alloys are classified into non-hardenable and hardenable alloys; hardenable alloys can be heat-treated to reach higher mechanical properties. Precipitation hardening enables the precipitation of a second phase into the aluminium matrix. Consequently, in the HAZ, mechanical properties may be lower than those in the base metal, due to the solubilization or coarsening of the intermetallic phases. For instance, Wang et al. [5] studied the microstructures and the mechanical properties of a dissimilar MIG weld of alloys 6xxx and 7xxx using the filler metal ER5356. The authors found a difference in the mechanical properties of the alloys’ HAZ that depends on the different strength mechanisms. The thermal cycles may cause the local dissolution of the intermetallic phases.

The absence of precipitate-forming elements into the non-heat-treatable alloys may be a positive feature for weldability: intermetallic phases permit precipitation hardening [4] but may lead to hot cracking during the welding. However, heat-treatable alloys are used for their higher mechanical properties concerning non-treated or annealed compositions.

Interestingly, pure aluminium has become essential in different industries because of its properties. For this reason, various publications focus on commercially pure aluminium alloys. Alloy AA1070, in particular, is a wrought and non-heat-treatable alloy; its mechanical properties can be successfully increased by cold or hot working. For instance, Al Quasaab [6] assessed that after cold working, ultimate tensile stress in alloy AA1070 might change from almost 30 to 160 MPa (30% and 80% reduction in thickness, respectively). At the same time, the elongation at rupture drops from 13% to 4%.

Ashtiani et al. [7] used a constitutive model for the hot deformation behaviour of alloy AA1070 to predict the flow stress with different initial grain sizes in the hot working process. Barekatain et al. and Shankar et al. [8,9] successfully welded alloy AA1050 to the commercially pure copper sheet through FSW. Lu et al. [10] focused on welding behaviour in dissimilar joint AA1060-AISI 304. Jayakrishnan et al. [11] assessed the microstructure for commercially pure aluminium sheets welded via flux-bounded-TIG, focusing on welding parameters and flux particle size, finding that penetration depth increased for a smaller powder particle size.

Edge preparation before welding is another essential feature and depends on the metal’s thickness. The edge preparation affects the quality of the welding process and facilitates weld penetration, as studied by Singh et al. [12]. Standard BS EN ISO 9692-3:2016, in Europe, regulates the joint preparation to ensure an accurate edge preparation. Despite that, the chamfering may be avoided if the sheet thickness is less than 4 mm.

In the publication by Akkurt et al. [13], authors studied the effect of the cutting process on the surface microstructure and hardness of both pure aluminium and aluminium alloy AA6061. They found that the heat-based cutting processes cause microstructure modification near the cut surface, while cold deformation may occur after mechanical cuttin AA1070 AA1070g.

Singh et al. [12] studied various cutting processes for cutting commercially pure aluminium sheets, finding that the most detrimental process in terms of edge preparation is the plasma cutting process.

As for the plasma cutting, literature research conducted by Stournaras et al. [14] highlights that laser power and cutting speed plays the most crucial role in the cut quality of aluminium sheets. Furthermore, a high laser power causes heat-affected zones into the sheets, while high gas pressures result in effective materials removal.

Shear cutting is one of the most common processes for cutting metal sheets. Stournaras et al. [14] studied the shearing of alloy AA6014 and noticed that slivers occur due to various parameters, such as the blank holders’ pressure and the adhesive behaviour of aluminium alloys. Moreover, Al Quasaab [6] investigated the shear angle during the orthogonal cutting, finding that suitable models may predict the shear angle. Even a waterjet can cut aluminium alloys. Al Qassab [6] also focused on the waterjet cutting process, underlining that the final sheet surface has two types of textures: a smooth surface on the upper side of the sheet and a rough one on the bottom surface of the sheet.

Filler metal and surface preparation play an essential role in fusion welding, mainly because they affect the mechanical and microstructural behaviour of the joint. For instance, a filler metal similar to the base metal, ER1100 for alloy AA1070, may preserve the electrical and corrosion resistance. On the other hand, it is possible to adopt other filler metals, such as ER4043 or ER5356, to increase the mechanical properties of the joint.

In this work, aluminium AA1070 sheets were cut using three cutting processes (shearing, waterjet and plasma) and then welded. The welding processes adopted in this work are TIG and MIG, using the filler metals ER5356 (rod shape for TIG welding) and ER4046 (wire shape for MIG welding), respectively. The obtained sheets were mechanically tested to assess the effectiveness of the welding process through tensile tests, hardness and micro-hardness measurements; the fracture surfaces and microstructures were studied to highlight a relation between the cutting process and the tightness of the welding seam.

## 2. Materials and Methods

In this study, commercially pure AA1070 H16 aluminium alloy sheets were cut using three different cutting processes (shearing, water jet and plasma) and then joined using TIG or MIG welding. Filler metal ER5356 was used in the form of a rod to perform TIG welding, while filler metal ER4043 in the form of wire was adopted in MIG welding. An external supplier performed cuts and welding processes. The total dimensions of the welded sheets were 250 mm × 350 mm × ca. 3 mm; for each welded sheet eleven specimens for tensile tests and a small sample for micro-hardness and microstructural analysis were milled. The composition of alloy AA1070 and fillers ER4043 and ER5356 were measured using an optical emission spectrometer, GNR S7-MLP (G.N.R. S.r.l., Novara, Italy); compositions are reported in Table 1.

Cutting parameters are reported in Table 2, along with welding parameters. As for plasma cutting, criteria followed the BS EN ISO 9013:2017 standard. Since there are no standards for quality criteria of waterjet cutting, the same plasma standard was considered. Regarding shearing, standard VID 2906-2 was used.

Sheets’ edges were observed after the cutting processes via optical microscope (LEICA MEF4M, Leica Microsystems, Heerbrugg, Switzerland) and stereomicroscope (LEICA MS5, Leica Microsystems, Heerbrugg, Switzerland).

**Figure 1:**
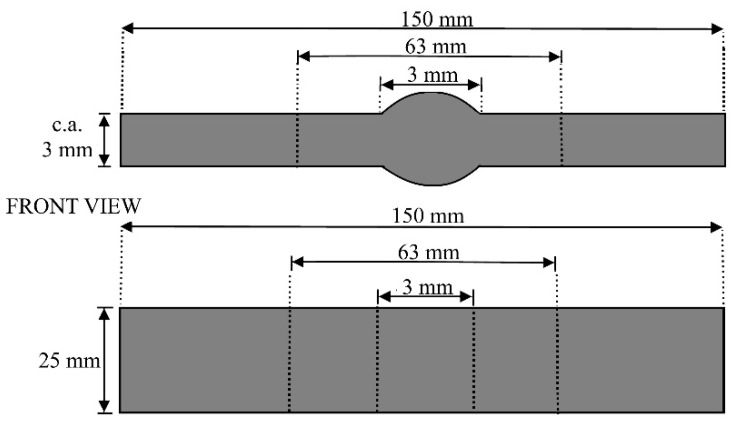
Drawing of specimens for tensile tests. Welding length: 3 mm; gauge length: 60 + weld length; thickness: ca. 3 mm; width: 25 mm; total length 150 mm.

**Figure 2:**
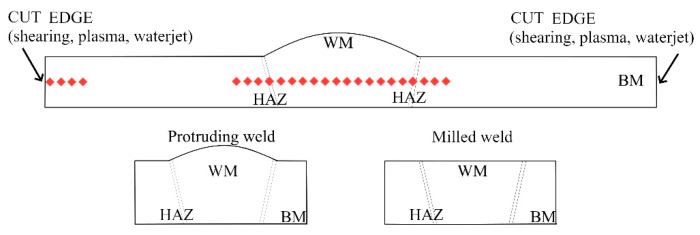
Drawing of micro-hardness measures path (red-diamond) along with BM, HAZ and WM and near the edges. Representation of the weld in the protruded and in the milled state.

Specimens for tensile tests were milled following the UNI EN ISO 4136:2012 standard (Figure 1): gauge length 120 mm, length 150 mm, width 25 mm, thickness 3 mm. Test parameters used were preload 50 N, test speed 8 N/mm^2^s^−1^ before yielding point R_p0,2_ and 0.008 N/mm^2^s^−1^ after yielding point R_p0,2_. Each welded sheet contained eleven specimens: five specimens had a protruding weld, while in the other six samples the weld was milled, as drawn in Figure 2. In fact, a protruded weld could be detrimental to the mechanical resistance, and it is commonly considered a weld-shape defect. Tensile tests were performed by a Zwick-Roell machine (Zwick-Roell BT1-FR100, Zwick Roell S.r.l., Genova, Italy).

**Figure 3:**
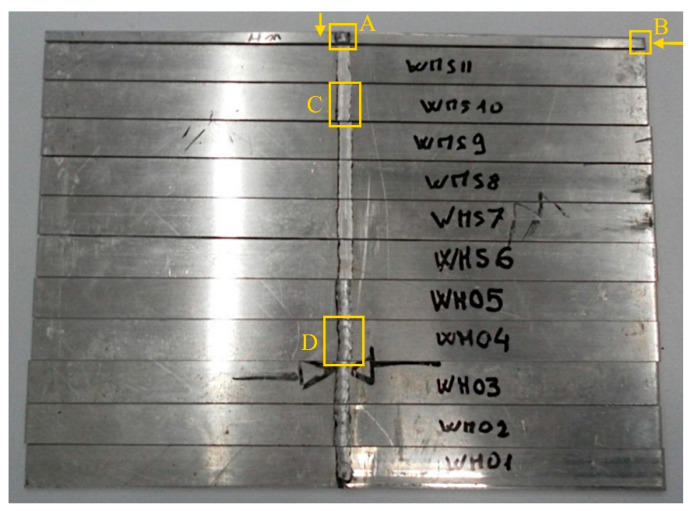
Sheets after waterjet cutting and MIG welding. The welded sheets were cut to obtain tensile test specimens and samples for microstructural observation and micro-hardness measures. A: Sample for weld observation and micro-hardness; the arrow indicates the side of observation. B: Sample for edge observation and micro-hardness; the arrow indicates the side of observation. C: Milled weld. D: Protruding weld.

Cross-sectional micro-hardness measurements were performed on the welded samples using a Vickers micro-hardness tester, LEICA VMHT (Leica Microsystems, Heerbrugg, Switzerland), as shown in Figure 2. The parameters applied were 200 gf and 15 s. The number of specimens and tests are reported in Table 3, while samples’ production in a single welded sheet is shown in Figure 3.

Specimens for micro-hardness evaluation were resin-mounted and polished through SiC paper from 180 up to 2400 grit, then polished on a cloth with colloidal silica and finally etched with HF for 20 s. Scanning electron microscope (SEM, Zeiss E.V.O. 15, Zeiss, Oberkochen, Germany) equipped with secondary and backscattered electrons detector (EDS, Oxford Ultim Max, Oxford Instruments plc, Abingdon, UK) was used to analyse the base metal, heat affected zones and the welded metals.

## 3. Results

### 3.1. Edges Preparation: Shearing

**Figure 4:**
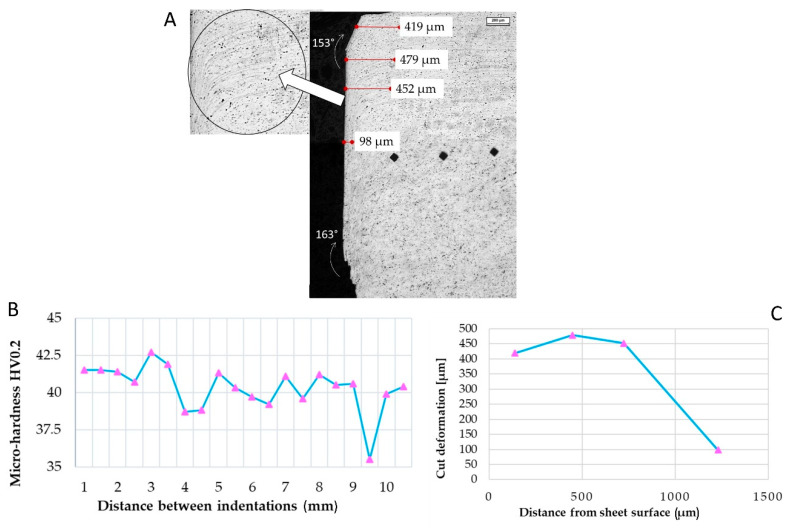
(**A**) Microscope image of the shearing cutting trace on AA1070 sheet. (**B**) Distortions in the first 1200 µm were documented. (**C**) Micro-hardness measured along the first 10 mm starting from the cut edge. Marker: 200 µm.

**Figure 5:**
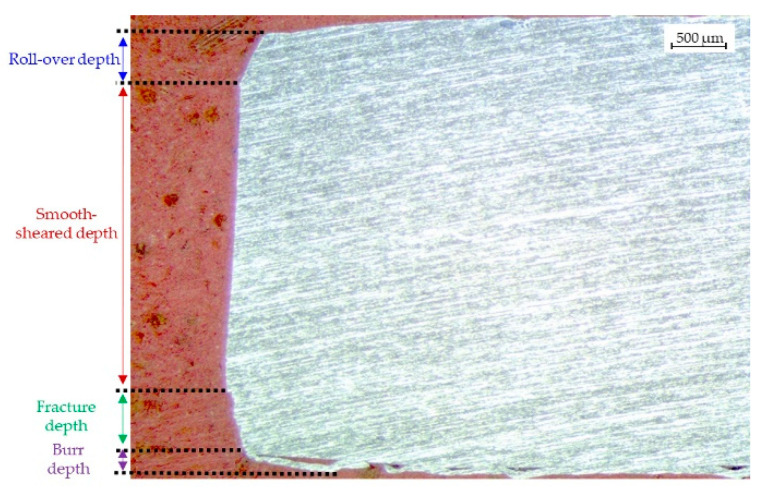
Different zones of the edges after shearing.

The aluminium sheets after the shearing process appear to have almost linear edges, as clearly noticeable from Figure 4A and Figure 5. The sheet microstructure is oriented along the rolling direction (horizontal direction); after the chemical etching, the distortion of the microstructure at the edges appears evident.

Some microstructural measures were performed through image analysis software (LEICA QWin, version 3.5, Leica Microsystems, Heerbrugg, Switzerland) to evaluate the microstructural distortions. Shearing caused damage to the sheet’s edges and material removal. Deformation angles were 27° (top sheet) and 17° (bottom sheet), while distortions decreased from 419 on the top to 98 µm in the middle of the sheet (Figure 4B).

Micro-hardness measured along the edge after shearing did not show an increase caused by plastic deformation near the edge; the micro-hardness resulted almost constant along with the sheet (Figure 4B).

### 3.2. Edges Preparation: Water Jet

**Figure 6:**
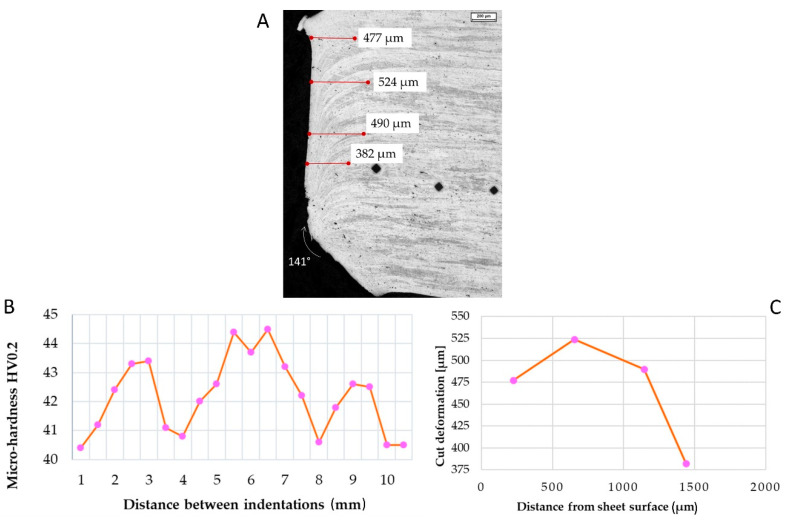
(**A**) Microscope image of the water jet cutting trace on AA1070 sheet. (**B**) Distortions in the first 1500 µm were documented. (**C**) Micro-hardness measured along the first 10 mm starting from the cut edge. Marker: 200 µm.

As for the water jet (Figure 6A), the initial distortion remained almost constant, with a measured value of 382 µm in the middle of the sheet (Figure 6C). The bottom part of the sheet showed an intense deformation and material removal; an angle of 141° was measured. Only short variations in micro-hardness were documented (Figure 6B).

### 3.3. Edges Preparation: Plasma

**Figure 7:**
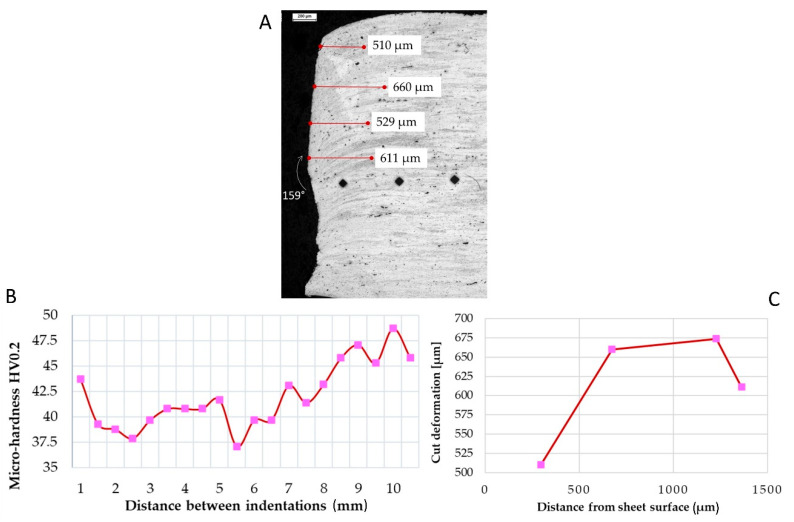
(**A**) Microscope image of the plasma cutting trace on AA1070 sheet. (**B**) Distortions in the first 1400 µm were documented. (**C**) Micro-hardness measured along the first 10 mm starting from the cut edge. Marker: 200 µm.

Plasma cutting (Figure 7A) caused higher specimen edge and microstructure orientation distortions. The distortion near the surface was 510 µm and at the mid-thickness 611 µm (Figure 7B).

A slight increase in micro-hardness was detected near the sheet’s edge (Figure 7B).

Furthermore, significant damage was caused during the cutting process, with material removal in the lower half of the sheet, with a measured angle of 159°, while 180° was expected for a plain surface. Almost 100 µm of material was removed during the cut, causing a potential lack in penetration of the welding seam.

In the first 8 mm, the micro-hardness resulted lower, with a minimum of 37.5 HV, then micro-hardness started increasing up to 45 HV.

### 3.4. Weld Microstructures

Weld microstructures were observed through an optical microscope and SEM.

**Figure 8:**
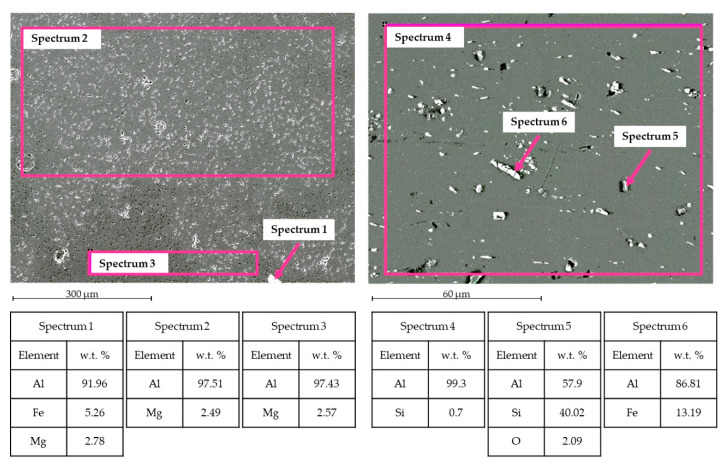
TIG welding analysis. Left side: SEM image and EDS in WZ. Right side: SEM image and EDS in HAZ.

Figure 8 shows SEM-EDS analysis for TIG welding. In the WM, Al and Mg were observed, as expected, in the filler metal ER5356. Some intermetallic phases were observed, for instance, the phase Al-Fe-Mg [15] in Spectrum 1. In the HAZ zone, Al-Si and Al-Fe intermetallic phases were noticed [16].

**Figure 9:**
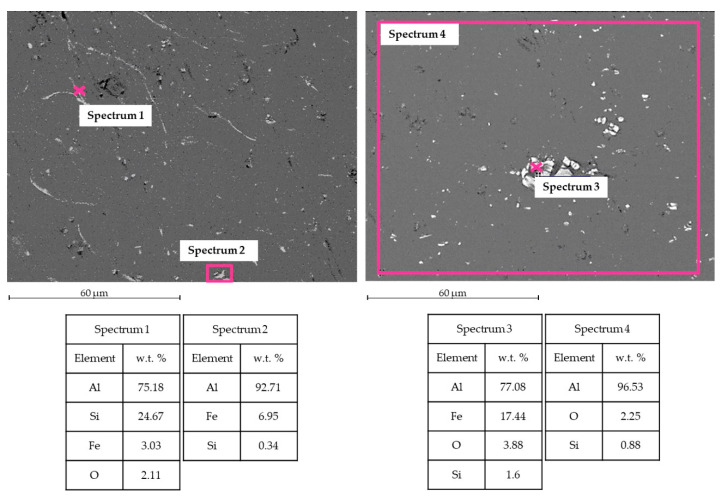
MIG welding analysis. Left side: SEM image and EDS in WZ. Right side: SEM image and EDS in HAZ.

Figure 9 shows SEM-EDS analysis for MIG welding. Into the WM, it was possible to observe the presence of Al and Si, typical elements for the filler metal ER4043. Some intermetallic phases are found; for instance, in Spectrum 1 and Spectrum 3, the phase Al-Fe-Si is in the form of acicular phase β and polygonal phase α [17]. Even Al-Si and Al-Fe phases occur in the HAZ zone.

**Figure 10:**
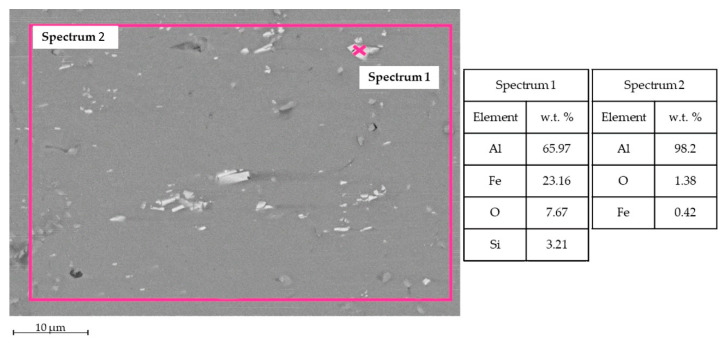
Base metal (BM) AA1070 H16.

The base metal contains a certain amount of Si and Fe, as shown in Figure 10. It is well known that, if Fe exceeds the solid solubility limit of 0.05 wt %, it forms intermetallic phases Al-Fe [18]. Due to the H16 starting structure, these phases show an elongated shape.

### 3.5. Tensile Tests and Fractography

Tensile tests were performed on samples shown in Figure 1 and Figure 3.

**Figure 11:**
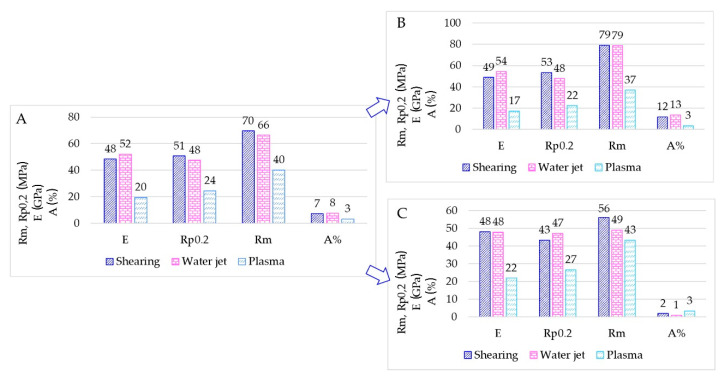
(**A**) Overall bar chart for TIG-welded sheets. (**B**) Bar charts for as-welded samples. (**C**) Bar charts for milled samples.

Results for TIG welding are summarized in Figure 11 through bar charts. The bar charts represent the average tensile properties measured (Figure 11A), the average tensile properties for the specimens as-welded (Figure 11B) and finally, the average tensile properties for the specimens having the milled weld (Figure 11C).

The average elongation at rupture A% in TIG-welded specimens is affected by the very low A% in the milled specimens. Mechanical properties observed (Rm, tensile strength; R_p0,2_, yield strength) for sheared and water jet cutting appear similar.

**Figure 12:**
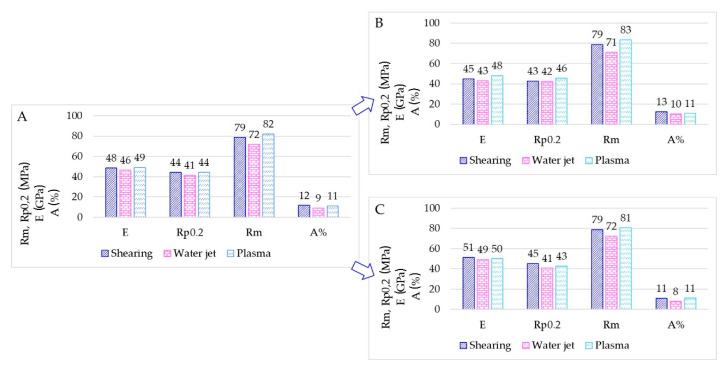
(**A**) Overall bar chart for MIG-welded sheets. (**B**) Bar charts for as-welded samples. (**C**) Bar charts for milled samples.

The average mechanical values of MIG-welded specimens are shown in Figure 12. Mechanical properties observed appear similar for each kind of sheet edge preparation, and the A% appear similar with or without the weld milling.

**Figure 13:**
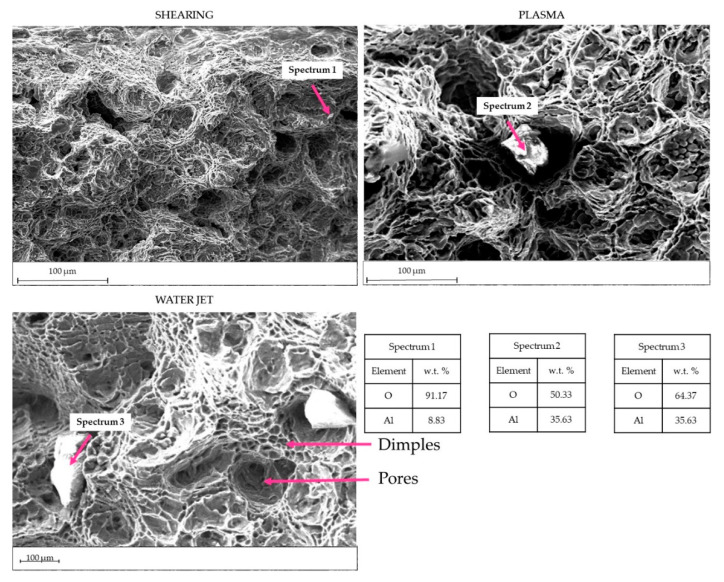
Surface fractures in three TIG-welded tensile specimens. Oxides were detected of variable sizes.

To assess these hypotheses, SEM observations were performed. The fracture surfaces were observed using SEM (Figure 13). Ductile fractures were noticed in all the specimens; all fractures were located in the welded part. Some welding appears non-penetrating along the entire thickness of the sheet. Furthermore, the weld seams appear rich in gas porosities [19] and in dendrites that negatively affect the mechanical strength. A high amount of oxide scale was observed, particularly in the water jet and plasma specimens (Figure 13—Spectrum 2 and Spectrum 3).

### 3.6. Weld Micro-Hardness

**Figure 14:**
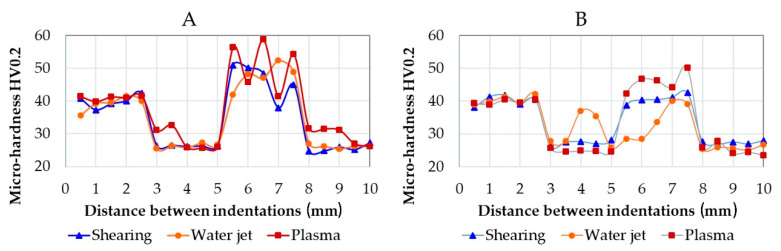
Graphical representation of the micro-hardness measured in TIG (**A**) and MIG (**B**) specimens.

The WM has a dilution ratio that depends on the welding process. WM comprises AA1070 base metal and ER5356 filler metal for TIG welding and AA1070 and ER4043 for MIG welding. The micro-hardness in the welded samples was measured as drawn in Figure 2, and results are shown in Figure 14. The different dilution ratios may affect the average micro-hardness. Furthermore, in the WM, the micro-hardness is subjected to a certain variation caused by defects or hardening phases. In TIG welding, specimens’ plasma-cuts show some inadequate penetration and porosities. This behaviour causes a variation in the micro-hardness measured (red-square marker in Figure 14A).

Moreover, porosities inside the weld cause a decrease in hardness near the BM values. On the contrary, in MIG welded specimens, specimens after water jet cutting (orange-bullet marker in Figure 14B) show low micro-hardness in WM. This behaviour is in line with the mechanical results shown in Figure 12 (the worst in MIG welding specimens).

Overall, higher micro-hardness was noticed in TIG welding with an average value of 48 HV0.2, while in MIG welding, the average value was 40 HV0.2. This result is, in turn, affected by the filler metal. In TIG welds, the micro-hardness in WM is higher than the micro-hardness in the BM; on the contrary, in MIG welds, the micro-hardness resulted similar in the BM and the WM. Micro-hardness values are average values depending on the intermetallic phases. Considering that the number of intermetallic phases is low in alloy AA1070, hardness in BM is almost the hardness of the α-Al phase: ca. 40 HV0.2. Furthermore, the micro-hardness in the α-Al depends on the alloying elements dissolved in the solid solution [20]. Intermetallic phases detected may affect the average values: α-AlFeSi and β-AlFeSi are respectively ca. 883 HV and 765 ([21]), Al-Mg ca. 48 HV [22], Al-Si ca. 80 HV [20].

## 4. Discussion

Sheet preparation before welding was conducted by three different cutting processes. Mainly, shearing is a widespread process. Yousefi et al. in [23] demonstrated that welded metal is formed on the tool at high-speed cutting, causing a material flow on the machined surface. The weld metal formed on the tool edge causes an increase in the surface roughness. On the other hand, no welded metal is formed at a very high cutting speed, and the surface roughness is low.

Conversely, various authors studied sheets’ shearing, such as Hambli et al. in [24] and Tekiner et al. [25]. Mainly, they highlight four different zones after the shearing, as shown in Figure 5; the clearance between the punch and the die will affect the precision of the sheet shape and extension of these zones.

The ideal cutting condition requires the lowest possible energy and the higher quality of the blanked sheet. The punch penetrates the material during the shearing, and the material is pulled down, creating the roll-over [26].

In terms of weldability, this configuration may affect the filler metal penetration, causing inadequate welding penetration during the welding process. On the other hand, despite the sheet edge shown in Figure 4 not being wholly planar, there was a proper welding penetration in both the welding processes (TIG and MIG). After TIG, specimens prepared by shearing resulted in the best mechanical values (along with water jet). On the other hand, mechanical tests showed similar behaviour for shearing-cut specimens and plasma-cut specimens after MIG processing.

Krinninger et al. [27] underlined that aluminium alloys’ adhesive tendency (galling) is possible. Hence, the cutting surface in Figure 4 and Figure 5 do not display the galling, while the cutting surfaces result in being relatively planar.

After the shearing cut, only the first 98 µm of the material was distorted in the middle of the sheet. For that reason, micro-hardness measures in the middle of the sample resulted in constant values.

Fracture surface analysis highlights a good penetration of the welding in specimens after shearing. Fractures happen in the welding seams; in fact, welding seams result in a consistent amount of oxides and dendrites that negatively affect mechanical strength. The excellent preparation of the surfaces after shearing permits a good penetration of the filler metal during both MIG and TIG welding, without lack in penetration.

Waterjet cutting is another cutting process that may be used in aluminium alloys. Chithirai et al. in [28] demonstrated a linear relationship between the waterjet parameters (pressure, abrasive mass flow rate, traverse speed and distance from the nozzle) and the surface roughness of aluminium. Water pressure has a higher influence: an increase in water pressure causes a decrease in the aluminium surface roughness. As the sheets are thin, the roughness was not measured; on the other hand, it may be assumed low [29]. In fact, from metallographic observation, it seems that the water pressure was high enough to warp and remove material (Figure 6A), and this behaviour represents a possible shape error during the water jet cutting [30,31]. The high deformation angle in the lowest part of the sheet after the cutting process (up to 40°) causes welding difficulties, with a lack of penetration for both types of welds, penalizing the weld-milled samples in terms of mechanical resistance. After milling the weld, the average strength Rm in specimens drops from 79 to 49 MPa in TIG welds. On the other hand, R_m_ remains constant in MIG specimens. Incorrect cleaning of the sheet surfaces before welding causes the oxide scales in the WM. These scales may affect the mechanical resistance of the welding. A high amount of oxide was noticed in TIG specimens (Figure 14). In this sense, oxide contamination could affect the mechanical tightness, as noticed after the tensile tests.

The variation in micro-hardness inside the WM was caused by the high amount of gas porosities found in the weld pool, as observed in the surface fracture analysis in Figure 14. The reduction in the micro-hardness from the BM to the HAZ is attributable to the intermetallic Al-Fe coalescence and the grain coarsening.

In [32], Wang et al. studied the kerf taper in aluminium alloy 6061, arguing that reducing the cutting speed causes the reduction of the kerf taper. Slow cutting speeds can produce a reverse taper, as seen in Figure 6, where the cut’s kerf width is wider at the bottom than at the top. The jet stream removed more material at the bottom of the sheet than at the top. The reverse taper can also occur when cutting very soft materials [33]. On the other hand, literature highlights that multipass cutting [34] may increase surface finishing by obtaining flat surfaces.

From the literature, plasma cutting is considered the worst in terms of microstructural modification; moreover, heat-affected zones may develop if plasma power is too high [12]. Figure 7 shows the sheet microstructure after the plasma process. High microstructural distortions were noticed in the first half-width of the sheet. A consistent material removal was observed in the lowest part of the sheet, with a measured angle of 159° (21° of deformation in respect to the planar surface). With regards to micro-hardness, the first indentation (near the sheet edge) resulted in ca. 44 HV0.2, probably caused by strain hardening, then the micro-hardness decreased. In the first 7 mm, a slight heat-affected zone was noticed, micro-hardness decreased to 37 HV0.2; away from the edge, the micro-hardness increased up to 45–47 HV0.2.

Overall, plasma cut sheets present the lowest mechanical properties after TIG-welding; although the A% appear similar for all specimens having the milled weld, E, R_m_ and R_p0,2_ show a marked difference. E and R_p0,2_ are mainly half of the values measured in shearing and waterjet specimens. The plasma cut seems to affect the TIG welding behaviour causing poor mechanical tightness. Conversely, specimens welded by the MIG process have E, R_m_ and R_p0,2_ doubled. According to these results, it appears evident that the plasma cut process did not affect the possible tightness of the welding. Mechanical results depend only on the welding process and filler metal adopted. In fact, in TIG specimens, large oxide scales were detected in the surface fractures, as shown in Figure 14. In MIG welding, the filler metal ER4043 gave higher castability overcoming the flatness issues.

## 5. Conclusions

The present work studied the effect of the different cutting processes (shearing, water jet and plasma) on welded joints’ microstructures and mechanical properties. After TIG and MIG welding, the AA1070 aluminium alloy welded sheets were analysed in micro-hardness Vickers, tensile tests and microstructural observations.

The following results were argued.

Near the edges, different cutting processes caused different effects on both microstructures and micro-hardness measured. Plasma cutting affects the micro-hardness near the edges: the first micro-hardness value was slightly higher (ca. 40 HV0.2), while in the next 7 mm, the plasma effect occurs, resulting in a HAZ (see Figure 7).

The different cutting technologies seem only to affect the welding behaviour in TIG welding. TIG-welded sheets present lower elongation at rupture, especially in samples having the milled weld after plasma cutting. Plasma-cut and TIG welded sheets showed the worst mechanical behaviour. Conversely, the best mechanical behaviour was obtained with plasma-cut and MIG welded sheets. Both welding techniques coupled to water jet or shearing cuts provided similar mechanical properties. These results indicate that the welding process did not affect the mechanical properties of samples if the sheet surfaces resulted in a planar shape.

Conversely, after plasma cutting, more than half width resulted in a non-planar shape, with an angle of 21°. This behaviour may affect the welding tightness after TIG welding, where both torch and filler rod inclinations are essential to make a good weld joint. On the other hand, the filler metal in the form of wire is already in the MIG torch, making the process easier.

Micro-hardness along the welds clearly shows a decrease in the HAZ’s hardness and an increase in the WM. Overall, BM presented similar micro-hardness values to WM in MIG-welded specimens, where ER4043 filler metal was used. On the other hand, a difference in micro-hardness was detected in TIG welded specimens, where ER5356 filler was used. Intermetallic phases Al-Fe and Al-Fe-Mg contributed to increasing the WM hardness.

In conclusion, sheets welded by MIG welding present similar average values in as-welded samples and with milled-weld samples. Only slight differences were noticed between the preparation of the different sheets. On the other hand, sheet preparation seems to affect mechanical properties in TIG welding. In particular, plasma cut sheets TIG welded showed low mechanical strength and elongation at rupture, evidencing a flaw in weld penetration. This behaviour may be due to the lack of surface flatness: in fact, almost the half-width of the sheet (Figure 7) resulted in non-planar, limiting the filler penetration in TIG welding mode.

## Figures and Tables

**Table 1:** Material compositions (wt %) measured by an optical emission spectrometer.

Materials/Elements	Si	Mg	Mn	Fe	Al + Trace Elements
AA1070	0.142	0.002	0.001	0.225	Bal.
ER 5356	-	2.170	0.010	0.298	Bal.
ER 4043	5.900	-	-	0.400	Bal.

**Table 2:** Cutting and welding parameters.

**Plasma**				
Current flow rate (A)	Cutting speed (mm∙min^−1^)	Plasma/Shield	Torch-to-work Distance (mm)	
50	1500	Air/Air	2.5	
**Waterjet**				
Pressure (MPa)	Distance to work piece (mm)	Abrasive type	Abrasive feed rate (g/min)	Cutting Speed (mm∙min^−1^)
350	2	Garnet 80 mesh	300	1500
**Shearing**				
Sheet orientation angle (°)	Blank holder clearance (mm)	Edge radius (µm)	Lubricant	
0	<0.5	50	Oil	
**TIG welding**				
Shielding gas	Welding speed (mm∙min^−1^)	Current (A)	Filler diameter (mm)	
Argon	ca. 300	Max. 150	2.4	
**MIG welding**				
Shielding gas	Welding speed (mm∙min^−1^)	Current (A)	Filler diameter (mm)	
Argon	ca. 495	Max. 120	1.6	

**Table 3:** Specification for specimen studied/tested.

Specimens Realized			Specimens Details
#2 sheets (ca. 175 × 250 × 2.9 mm) cut by shearing and welded	MIG	(1) sample for micro-hardness after MIG welding; (11) samples for tensile tests *.	Micro-hardness near the edge of sheets (3 samples, one for each cut); Micro-hardness along the weld, passing from the heat-affected zone HAZ to the base metal BM to the welded metal WM. 66 Tensile tests samples. For each type: 5 in as-welded condition; 6 with milled weld.
	TIG	(1) sample for edge analysis; (1) sample for micro-hardness after TIG welding; (11) samples for tensile tests *.	
#2 sheets (ca. 175 × 250 × 2.6 mm) cut by plasma and welded	MIG	(1) sample for micro-hardness after MIG welding; (11) samples for tensile tests *.	
	TIG	(1) sample for edge analysis; (1) sample for micro-hardness after TIG welding; (11) samples for tensile tests *.	
#2 sheets (ca. 175 × 250 × 2.6 mm) cut by water jet and welded	MIG	(1) sample for micro-hardness after MIG welding; (11) samples for tensile tests *.	
	TIG	(1) sample for edge analysis; (1) sample for micro-hardness after TIG welding; (11) samples for tensile tests *.	

* Overall 6 specimens were tested having milled welds instead of 5 because possible inadequate penetration is expected (milled welding may present less-resistant areas).
